# Deciphering the Role of the *SREBF1* Gene in the Transcriptional Regulation of Porcine Adipogenesis Using CRISPR/Cas9 Editing

**DOI:** 10.3390/ijms252312677

**Published:** 2024-11-26

**Authors:** Mehmet Onur Aksoy, Adrianna Bilinska, Monika Stachowiak, Tatiana Flisikowska, Izabela Szczerbal

**Affiliations:** 1 Department of Genetics and Animal Breeding, Poznan University of Life Sciences, Wolynska 33, 60-637 Poznan, Poland; onur.aksoy@up.poznan.pl (M.O.A.);; 2 Chair of Livestock Biotechnology, School of Life Sciences, Technical University of Munich (TUM), D-85354 Freising, Germany

**Keywords:** adipogenesis, CRISPR/Cas9, isoforms, MSC, pig, *SREBF1*, SREBP1

## Abstract

Sterol regulatory element-binding protein 1 (SREBP1) is an important transcription factor that controls lipid metabolism and adipogenesis. Two isoforms, SREBP1a and SREBP1c, are generated by alternative splicing of the first exon of the *SREBF1* gene. The porcine *SREBF1* gene has mainly been studied for its role in lipid metabolism in adipose tissues, but little is known about its involvement, and the role of its two isoforms, in adipogenesis. The aim of the present study was to introduce a deletion in the 5′-regulatory region of the *SREBF1c* gene, considered crucial for adipogenesis, using the Clustered Regularly Interspaced Short Palindromic Repeats/CRISPR-associated protein 9 (CRISPR/Cas9) method. This approach allows for the evaluation of how inhibiting *SREBF1c* transcription affects the expression of other genes essential for adipocyte differentiation, particularly *PPARG*, *CEBPA*, *CEBPB*, *CEBPD*, *GATA2*, and *FABP4*. It was observed that disrupting the *SREBF1c* isoform had no effect on the *GATA2* gene but did result in a decrease in the expression of the *CEBPA* and *CEBPD* genes, an increase in the expression of *CEBPB*, and an inhibition in the expression of the *PPARG* and *FABP4* genes. These changes in gene expression blocked adipogenesis, as could be seen by the failure of lipid droplets to accumulate. Our results provide evidence highlighting the pivotal role of the *SREBP1c* isoform in the regulation of porcine adipogenesis.

## 1. Introduction

Sterol regulatory element binding proteins (SREBPs) are a family of basic helix–loop–helix leucine zipper transcription factors. They regulate lipid homeostasis by binding to sterol regulatory elements (SREs) in target genes. Two proteins, SREBP1 and SREBP2, are encoded by the two genes *SREBF1* and *SREBF2*, respectively. The SREBP1 protein is involved in the synthesis of fatty acid, phospholipid, and triacylglycerol, while SREBP2 is responsible for the regulation of cholesterol metabolism [1,2]. Two isoforms of SREBP1, called SREBP1a and SREBP1c, have been recognized. They arise from alternative transcription start sites and differ in their first exon [3]. The SREBP1c isoform is the predominant form expressed in in liver, adipose tissue, and skeletal muscle, whereas expression of SREBP1a has been found in the spleen, small intestine, heart, thymus, and proliferating cell lines [4,5]. SREBP2 is ubiquitously expressed in different tissues, especially in embryonic tissues, liver, and adipose tissue [6,7].

The SREBPs have been extensively studied in rodents, in which lipogenesis is regulated in both the liver and adipose tissue, while, in nonrodent mammals such as the domestic pig, only one lipogenic organ is active—the adipose tissue [8]. SREBP1 has been recognized as a key regulator of lipogenesis in pigs [9]. The *SREBF1* gene plays a role in fat metabolism and intramuscular fat content in pigs. It has been shown that the expression of the *SREBF1* gene correlates with the fat deposition rate in growing pigs [10]. It is also important for the regulation of fat deposition in muscle during the postnatal growth of pigs [11]. Higher expression levels of the *SREBF1* gene have been observed in the muscle tissues of fatty pig breeds compared to those in the leaner pig [12]. Polymorphisms in the *SREBF1* gene have been associated with fatness traits in pigs [13,14,15].

**Figure 1:**
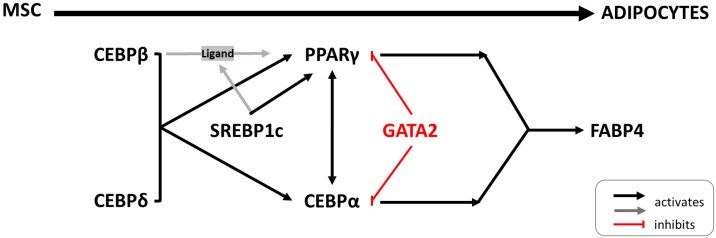
Simplified scheme of transcriptional regulation in adipogenesis, highlighting the transcription factors analyzed in this study. Adapted from [18].

SREBP1 is also involved in the regulation of adipogenesis. Initially, it was named “adipocyte determination and differentiation-dependent factor 1” (ADD1), which was later identified as the SREBP1c isoform [16,17]. The process of fat cells formation is controlled by a complex transcriptional network of many factors, of which proliferator-activated receptor γ (PPARγ) and members of the CCAAT-enhancer-binding protein (CEBP), such as C/EBPα and C/EBPβ, play a central role [18] (Figure 1). SREBP1 was identified as a proadipogenic transcription factor that induces PPARγ expression through the production of endogenous ligands [16]. The role of SREBP1 in adipogenesis has mainly been studied in mouse and human cells. There have been a number of studies suggesting that the *Srebf1c* isoform is the main transcription factor of adipogenesis [18,19,20]. The expression of this isoform increases during differentiation of the cultured mouse 3T3-L1 preadipocytes. On the other hand, studies in transgenic mice overexpressing SREBP1c have shown that adipocyte differentiation is inhibited, leading to lipodystrophy [21]. It has also been shown that the amount of white adipose tissue was not significantly decreased in mice with the disrupted *Srebp1c* gene [22]. Subsequent experiments on 3T3-F442A preadipocytes have revealed that the *Srebf1a* isoform is also a key regulator of transcriptional cascade during adipogenesis [23]. The loss of function of *Srebf1a* inhibits adipocyte differentiation. Research on *SREBF1* isoforms has also been extended to human cells: using the Simpson–Golabi–Behmel syndrome (SGBS) preadipocyte cell line as a valuable model for studying human adipocyte function [24]. RNA interference experiments in SGBS cells showed that while both SREBP1 variants were targeted, only SREBP1a expression significantly decreased [25]. The SREBP1a isoform was predominant in these cells, and SREBP1c was expressed at a low level. These studies show differences in the functioning of the SREBP1 transcription factor depending on whether cells or model organisms are used.

Data on the role of the *SREBF1* gene during adipogenesis in the pig are scarce, despite the importance of understanding the mechanisms controlling fat tissue formation in this key livestock species [26]. Adipose tissue grows by two processes: the generation of new adipocyte cells and the increase in the size of adipocytes [27]. Adipose tissue impacts neonatal survival, reproductive ability, postnatal growth, and meat production efficiency [28]. The pig has also been recognized as a better animal model for human obesity than rodents due to anatomical, physiological, and metabolic similarities with humans [29,30]. Regarding the role of the *SREBF1* gene during porcine adipogenesis, studies have only demonstrated that its expression levels increase progressively with the duration of differentiation [31,32]. Information on the role of the two *SREBF1* isoforms is also lacking.

Given the limited data on the role of the *SREBF1* gene and the significance of its two transcriptional forms in porcine adipogenesis, our study aimed to explore the pivotal role of this transcription factor in pigs. The revolutionary gene-editing tool CRIPSR/Cas9 has enabled the generation of gene-specific knockouts which can be used to study gene function [33]. To clarify the role of the *SREBF1* gene during adipogenesis in the pig, we generated a mesenchymal stem cells derived from adipose tissue (AD-MSC) with a targeted deletion in the *SREBF1* gene using the CRIPSR/Cas9 method. This deletion disrupts the 5′-regulatory region specific to the *SREBF1c* isoform while preserving the *SREBF1a* isoform, as the deletion was localized in an intron. Analysis of the transcriptional activity of the *SREBF1* gene in relation to other key genes that are important in adipocyte differentiation (*PPARG*, *CEBPA*, *CEBPB*, *CEBPD*, *GATA2*, and *FABP4*) allowed us to describe their expression patterns during differentiation. Understanding *SREBF1* activity provides valuable insights into the timing and sequential activation of key genes involved in adipocyte differentiation.

## 2. Results

### 2.1. Characterization of the 5′-Regulatory Region in Porcine SREBF1 and the Introduction of a CRISPR/Cas9-Mediated Deletion in This Region

**Figure 2:**
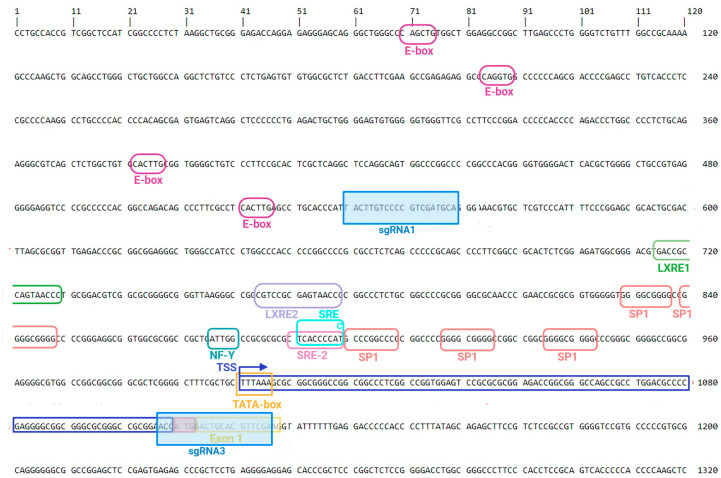
The 5′-regulatory sequence of *SREBF1c* with marked transcription factor binding sites and positions of two sgRNAs (sgRNA1 and sgRNA3 in blue frames) used to excise the proximal promoter region.

Since the promoter region of the porcine *SREBF1* gene has not been well characterized, we performed, as the first step, an in silico analysis of the 5′-regulatory sequence by identifying binding sites for known transcription factors for adipogenesis using DNA Star Lasergene (https://www.dnastar.com/software/ accessed on 10 January 2022). Several regulatory elements—such as TATA-box, SP1 elements, SRE/SRE-2, NF-Y, and LXRE1 motifs—were identified in the proximal 5′-regulatory region of the gene that encodes the *SREBF1c* isoform. To delete the proximal regulatory region harboring the majority of regulatory elements, four single guide RNAs (sgRNAs) were tested in different combinations, but, ultimately, two were selected for introducing cleavage sites in porcine mesenchymal stem cells derived from adipose tissue (AD-MSC) (Figure 2, Table S1).

**Figure 3:**
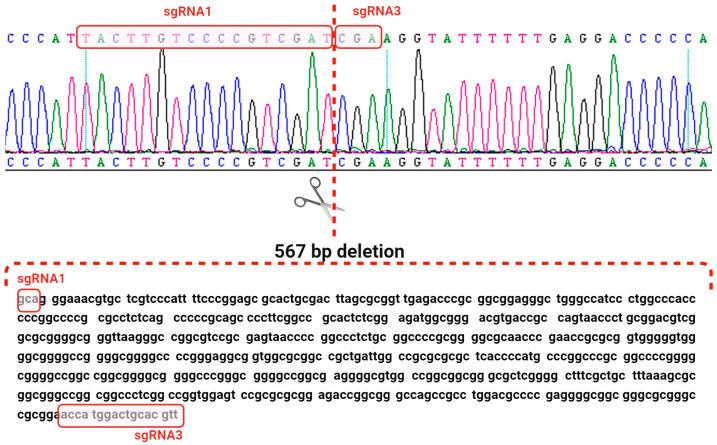
Sanger sequencing of the PCR product confirmed the deletion of 567 bp in the *SREBF1c* 5′-regulatory region. The position of the joined DNA fragments in the AD-MSC cells and the partial sequences recognized by sgRNA1 and sgRNA3 are shown.

**Figure 4:**
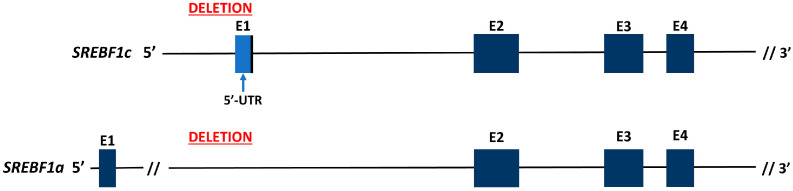
Location of the 567 bp deletion site in the genomic region covering *SREBF1a* and *SREBF1c* isoforms according to Sscrofa 11.1 assembly (NC_010454.4) chromosome 12 reference sequence. E1–E4: exons 1–4. Genomic position of the 567 bp deletion: 12: 60740868-60741434.

sgRNA1 and sgRNA3 were cloned into the pX330 plasmid, and AD-MSCs were transfected via nucleofection. The transfection efficiency of AD-MSCs, measured with a control plasmid (pmaxGFP Vector, Lonza, Basel, Switzerland), was approximately 40% (Figure S1). Single AD-MSC colonies post-transfection were genotyped by PCR, targeting regions overlapping the sgRNA binding sites, resulting in the identification of two colonies with deletions in the target gene (Figure S2). Subsequent Sanger sequencing of PCR products from these cells revealed a 567 bp deletion in the 5′-regulatory region of *SREBF1c* (Figure 3). In silico analysis of the porcine *SREBF1* gene sequence confirmed that this deleted fragment was located within intron 1 of the *SREBF1a* isoform (Figure 4). This approach enabled us to obtain modified AD-MSCs with a non-functional *SREBF1c* isoform while preserving an intact transcript of the *SREBF1a* isoform.

### 2.2. Characteristics of the Expression Profile of the SREBF1a and of SREBF1c Isoforms in Wide-Type Cells (AD-MSC_WT_) and in AD-MSC with Disrupted SREBF1c Gene (AD-MSC_DEL_) and Adipose Tissues

**Figure 5:**
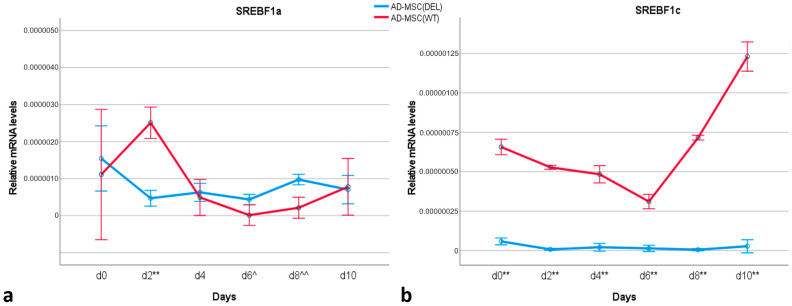
Relative transcript levels of *SREBF1a* (**a**) and *SREBF1c* (**b**) during subsequent days of adipogenesis. The error bars represent 95% confidence intervals. **: significantly higher in AD-MSC_WT_ than in AD-MSC_DEL_, *p* < 0.001; ^^: significantly lower in AD-MSC_WT_ than in AD-MSC_DEL_, *p* < 0.001; ^: significantly lower in AD-MSC_WT_ than in AD-MSC_DEL_, *p* < 0.05.

The relative transcript level of the *SREBF1a* isoform was similar in undifferentiated cells (day 0) for both AD-MSC_WT_ or AD-MSC_DEL_ (Figure 5a). After induction of adipogenesis, increased expression of this isoform was detected on day 2 in AD-MSC_WT_, with its level being significantly higher than in AD-MSC_DEL_ (*p* < 0.001). In days 6 and 8 of differentiation, a higher level of *SREBF1a* was observed in AD-MSC_DEL_ (*p* < 0.05 for day 6 and *p* < 0.001 for day 8). The analysis of the transcript level of *SREBF1c* in AD-MSC_WT_ indicated that there was an increase in expression from day 8 of differentiation (Figure 5b). As expected, no expression of this isoform was detected in AD-MSC_DEL_.

**Figure 6:**
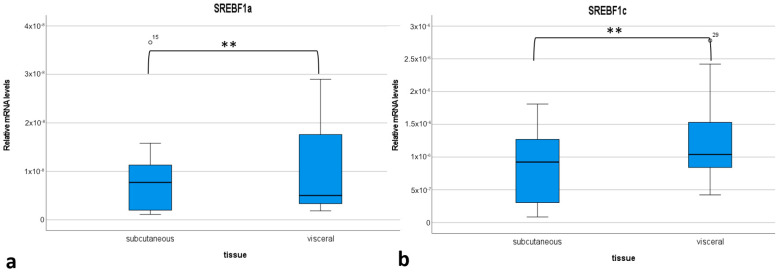
Relative mRNA levels of *SREBF1a* (**a**) and *SREBF1c* (**b**) isoforms in the subcutaneous and visceral adipose tissue of wild-type pigs. The boxplots show the lower quartile (lower edge of the box), median (black line dividing the box into two parts), upper quartile (upper edge of the box), outliers (dots), and whiskers (values inside the lower and upper quartiles). **: significant differences between groups, *p* < 0.001, Wilcoxon’s signed rank test.

Additionally, the expression of both isoforms of the *SREBF1* gene was analyzed in porcine adipose tissue to determine which isoform plays an essential role in the function of this tissue in pigs. Two adipose tissue were analyzed—subcutaneous and visceral. In both tissues, the expression of *SREBF1c* was significantly higher than in *SREBF1a* (Figure S3). Both transcriptional forms showed higher expression in visceral fat tissues (*p* < 0.001) (Figure 6).

### 2.3. Monitoring of Lipid Droplet Accumulation in AD-MSC_WT_ and in AD-MSC_DEL_

**Figure 7:**
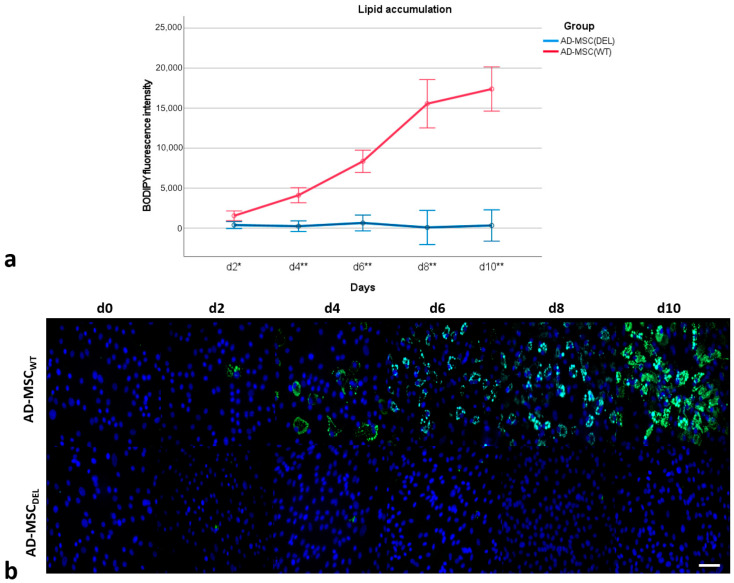
Measuring of lipid accumulation based on BODIPY 493/503 fluorescent intensity during successive days of adipocyte differentiation (**a**). Error bars represent 95% confidence intervals. D: day; **: significantly higher in AD-MSC_WT_ than in AD-MSC_DEL_, *p* < 0.001; *: significantly higher in AD-MSC_WT_ than in AD-MSC_DEL_, *p* < 0.05. Representative images showing adipocyte differentiation (**b**). Lipid droplets were stained with BODIPY 493/503 (green); the nuclei were counterstained with DAPI (blue). Scale bar: 50 µm.

The accumulation of lipids was monitored using BODIPY staining for ten days of adipogenesis (Figure 7). On day 0 of differentiation (MSC), no lipid droplets were observed. On day 2 (d2), differentiation of single cells was visible in both AD-MSC_WT_ and AD-MSC_DEL_, but the amount of lipid droplets was higher in AD-MSC_WT_ (*p* < 0.05). With day 4 of adipogenesis, a significant increase (*p* < 0.001) in lipid droplet accumulation was observed in AD-MSC_WT_, while cells with a disrupted *SREBF1c* isoform (AD-MSC_DEL_) did not accumulate lipid droplets and did not differentiate into adipocytes.

### 2.4. Expression of Key Adipogenic Genes in AD-MSC_WT_ and AD-MSC_DEL_

**Figure 8:**
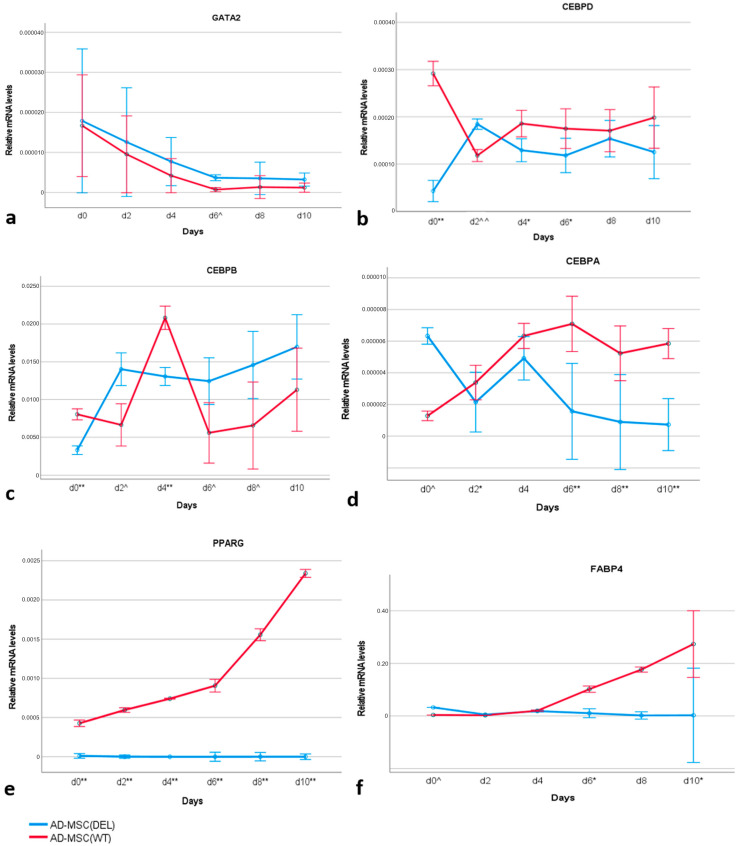
Relative transcript levels of *GATA2* (**a**), *CEBPD* (**b**), *CEBPB* (**c**), *CEBPA* (**d**), *PPARG* (**e**), and *FABP4* (**f**) during subsequent days of adipogenesis in AD-MSC_WT_ and AD-MSC_DEL_. Error bars represent 95% confidence intervals. **: significantly higher in AD-MSC_WT_ than in AD-MSC_DEL_, *p* < 0.001; *: significantly higher in AD-MSC_WT_ than in AD-MSC_DEL_, *p* < 0.01; ^^: significantly lower in AD-MSC_WT_ than in AD-MSC_DEL_, *p* < 0.001; ^: significantly lower in AD-MSC_WT_ than in AD-MSC_DEL_, *p* < 0.05.

The expression patterns of six genes important for adipogenesis were evaluated in AD-MSC_WT_ and AD-MSC_DEL_. These genes were selected as key transcription factors involved in adipogenesis (*PPARG*, *CEBPA*, *CEBPB*, *CEBPD*, and *GATA2*) and as a marker gene for adipocytes (*FABP4*).The *GATA2* gene was selected as an anti-adipogenic transcription factor, *CEBPD* and *CEBPB* as early genes induced during adipogenesis, *CEBPA* and *PPARG* as critical transcription factors in adipogenesis, and *FABP4* as a gene important for terminal adipocyte differentiation. *GATA2* was downregulated during adipocyte differentiation in both AD-MSC_WT_ and AD-MSC_DEL_, and its transcript level was not significantly different between these cell types apart from on day 6 (*p* < 0.05) (Figure 8a). The expression profile of *CEBPD* was very similar in AD-MSC_WT_ and AD-MSC_DEL_, with visibly higher expression in AD-MSC_WT_ on day 0 (*p* < 0.001), day 4 (*p* < 0.01), and day 8 (*p* < 0.01) (Figure 8b). *CEBPB* showed a characteristic peak of transcriptional activity on day 4 (*p* < 0.001) of adipogenesis in AD-MSC_WT_. *CEBPB* was the only gene to show a higher transcript level in AD-MSC_DEL_ (*p* < 0.05) than in AD-MSC_WT_ (Figure 8c). *CEBPA* was upregulated during differentiation in AD-MSC_WT_, and its transcript level was significantly higher in AD-MSC_WT_ than in AD-MSC_DEL_ on day 2 (*p* < 0.01), day 6, day 8, and day 10 (*p* < 0.001) (Figure 8d). Increasing transcript levels of *PPARG* and *FABP4* genes were observed during differentiation in AD-MSC_WT_ (Figure 8e,f). An increase in *PPARG* gene expression was observed over the subsequent days of examination, whereas *FABP4* gene expression was detected starting from day 6 of differentiation. No expression of either gene was found in AD-MSC_DEL_ (*p* < 0.001).

Summarizing this experiment (Table 1), we showed that the lack of activity of *SREBF1c* resulted in adipogenesis being blocked and in the *PPARG* and *FABP4* genes not being expressed. The expression levels of *CEBPA* and *CEBPD* genes were reduced. The modification did not cause major changes in *GATA2* gene expression—instead, an increase in the expression of the *CEBPB* gene was observed in the modified AD-MSC. Since PPARγ activation relies on lipid-derived ligands produced through SREBF1c activity, the absence of the *SREBF1c* transcript led to disrupted expression of *PPARG* and, consequently, its downstream target, *FABP4*. The disruption of the *SREBF1c* isoform affected the expression of genes acting upstream in the adipogenesis cascade, resulting in an altered expression pattern in AD-MSC_DEL._

## 3. Discussion

Adipocyte differentiation is governed by the complex action of many transcription factors. It has been well established that PPARγ is the master regulator of adipogenesis and that the members of the C/EBP family play critical roles in the normal course of this process. Studies of the importance of SREBP and their isoforms during adipogenesis have provided different results, and there are no data about the role of these isoforms in the formation of fat cells in the pig. In the present study, we used the CRIPSR/Cas9 genome editing technique to reveal the crucial role of the *SREBF1c* gene in adipogenesis in the pig. The study was performed on a well-established system of in vitro differentiation of mesenchymal stem cells into adipocytes [34,35]. Temporal changes in the transcript level of seven genes (*SREBF1*, *PPARG*, *CEBPA*, *CEBPB*, *CEBPD*, *GATA2*, and *FABP4*) were detected in both wild-type and modified AD-MSCs. The transcriptional profiles of the genes in AD-MSC_WT_ were similar to those observed during adipocyte differentiation of 3T3-L1 cells and to experiments performed previously on the porcine in vitro adipogenesis system [31]. As a negative regulator of adipogenesis, *GATA2* was downregulated during porcine adipogenesis. The murine *Gata2* mRNA level was also found to decrease very soon after the induction of adipocyte differentiation [36]. *Cebpb* and *Cebpd* are early transcription factors and so usually have the same profile during adipogenesis in mouse cell lines, with peaks in the early hours or days of differentiation, but then declining [37]. In our differentiation system, this type of profile was observed only for *CEBPB*. The expression of *PPARG* and *CEBPA* gradually increased over the subsequent days of differentiation, while the *FABP4* gene was upregulated in the final day of differentiation, which is in accordance with observations in other murine or porcine in vitro adipogenesis systems [35,38].

The analysis of the expression profiles of *SREBF1a* and *SREBF1c* in AD-MSC_WT_ showed that an increase in the expression of *SREBF1a* takes place in the early stages of differentiation, which is why *SREBF1c* is upregulated in the late stages of adipogenesis. The same trend was observed in 3T3-F442A cells where the expression of *Srebf1a* was found in the very early stage of induction, before *Srebf1c* [23]. However, it should be pointed out that the 3T3-F442A cell line shows very rapid adipocyte differentiation in vitro, taking only hours with the use of staurosporine or dexamethasone induction [39]. The entire differentiation process lasted six days. Our study was conducted on MSC derived from adipose tissue, which reflects a more physiological process, as this type of MSC has a higher adipogenic differentiation capacity [40]. Additionally, we also showed in this study for the first time the differences in the expression level of two isoforms of the *SREBF1* gene in subcutaneous and visceral adipose tissue in pigs: *SREBF1c* was the predominant isoform expressed in these porcine adipose tissues. This is in accordance with data on humans showing that this isoform has greater expression in different adipose tissue depots than does *SREBF1a* [41].

The use of the CRISPR/Cas9 editing method to generate a deletion in the *SREBF1* gene, so as to disrupt the *SREBF1c* isoform but not the *SREBF1a* isoform, allowed comprehensive analysis of the expression pattern of genes that encode the crucial transcriptional factors and marker proteins for adipogenesis in the absence of *SREBF1c*. This modification had no effect on *GATA2* gene expression in AD-MSC_DEL_. This may be due to the fact that this factor is expressed only in the very early stages of adipogenesis (before expression of the *SREBF1c* isoform) and its downregulation is necessary to induce differentiation into adipocytes [31,36]. The *CEBPD* gene expression pattern was also similar in both AD-MSC_DEL_ and AD-MSC_WT_, which may indicate that there is no direct link between *CEBPD* and *SREBF1c* transcriptional factors. An interesting observation was made for the *CEBPB* gene, which was the only one to show a higher expression in AD-MSC_DEL_ than in AD-MSC_WT_. It is known that C/EBP transcription factors may regulate the SREBP1c gene expression during adipogenesis. For example, C/EBPβ can directly regulate SREBP1c by binding to its promoter region, and mice lacking C/EBPβ have reduced SREBP1c levels in adipose tissue [42]. It has also been shown that SREBP-1c can directly transactivate the C/EBPβ promoter [43]. Another study has shown that C/EBPβ phosphorylation promotes the expression of *Srebf1a*, which induces the expression of other adipogenic genes like *Pparg*, *Cebpa*, and *Srebf1c* [23]. So far, there have been no data on the impact of the lack of *SREBP1c* expression on *CEBPB* expression. It can be assumed that if the *SREBP1c* transcript is missing, a compensatory mechanism could lead to increased expression of the *CEBPB* gene. Our observation that *CEBPB* expression is increased in AD-MSCs lacking *SREBF1c* expression may suggests that *CEBPB* may respond to the loss of *SREBF1c* to maintain the adipogenic process, possibly helping to stabilize adipocyte development in the absence of this key isoform. Decreased expression was observed for the *CEBPA* gene, and complete inhibition of the expression of the *PPARG* and *FABP4* genes in AD-MSC_DEL_ was noted. Since *SREBF1* expression is essential for providing lipid ligands to PPARγ [16], the disruption of *SREBF1c* transcription can block the expression of *PPARG* itself and its downstream target, *FABP4* [44]. Given the regulatory relationship between PPARγ and C/EBPα [19], the absence of *SREBF1c* may consequently lead to reduced *CEBPA* expression. The study conducted by the authors of [23] showed that the order of expression of adipogenic genes during adipogenesis using 3T3-F442A cells is as follows: *Cebpb*, *Srebf1a*, *Pparg2*, *Cebpa*, *Srebp1c*, and *Fabp4*. The expression pattern observed in our in vitro model of adipogenesis indicates that *CEBPA* is activated before *PRRAG* and *FABP4*. Since transcriptional regulation of adipogenesis is governed by the expression of the two main players *PPARG* and *CEBPA* [45], its absence blocks adipocyte differentiation; this was observed in our system as failure of lipid droplets to accumulate.

It is possible that the modification introduced in the *SREBF1c* gene locus may also affect the expression of other genes. A limitation of our study is its focus on a selection of genes specifically associated with adipogenesis and mature adipocytes. To address this, future research should employ RNA-Seq analysis, which would provide a comprehensive dataset and deeper insights into gene expression changes across the transcriptome.

Our studies have demonstrated the effectiveness of the CRISPR/Cas9 method as a gene-editing tool for examining gene functions. By generating MSCs with a deletion in the regulatory region of the *SREBF1* gene, we were able to identify the critical role of the *SREBF1c* isoform in adipogenesis. To the best of our knowledge, this is the first experiment of this type in the pig. To date, such experiments have been conducted only in other organisms. *Srebp*-1c knockout mice have been used to investigate the upstream and downstream regulatory mechanisms of SREBP-1c in vitro [46]. Knockout of the *SREBF1* gene by the introduction of a mutation (a 1bp deletion to produce a termination codon) using CRISPR/Cas9 has previously been conducted for zebrafish [47], where it gave insight into the biology of *SREBF1* in the fatty metabolism and musculoskeletal functioning of the zebrafish. In our study, we introduced a mutation to this gene, in the form of a large deletion of the 5′ regulatory region. A similar approach has been used to introduce large deletions in the porcine *SRY* gene for study causes of sex reversal in gene-edited pigs [48] and a 93-bp deletion in the 3ʹ-untranslated region (UTR) of the TNFα gene to generate a porcine Crohn’s disease model [49]. This shows how advances in CRISPR/Cas9 technology have strongly impacted swine research, enabling precise genetic changes that could improve pig breeding, increase disease resistance, and develop biomedical models [50,51,52].

Modifying porcine MSCs with CRISPR/Cas9 technology shows promise in creating animal research models for human diseases, including obesity. This technology has been used to silence genes crucial for adipocyte differentiation, such as *PPARG* and *FKBP5* [53], as well as to target three genes (*SOCS3*, *DUSP1*, and *SIK1*) that are upregulated in the adipose tissue of patients with nonalcoholic fatty liver disease (NAFLD) [54]. These experiments were performed on preadipocytes derived from adipose tissue and human adipose-derived mesenchymal stem cells (hADMSCs), respectively. Researchers have also developed human brown-like cells (HUMBLE) by activating the *UCP1* gene in human white preadipocytes using CRISPR/Cas9 technology [55]. Additionally, there are examples of using ribonucleoproteins (RNPs) consisting of the Cas9 protein and sgRNA to disrupt the *NRIP1* gene in mouse and human progenitor cells ex vivo prior to their differentiation into adipocytes. This approach enabled the generation of adipocytes with beige characteristics [56].

Building on these technologies, it is expected that in the future, pigs could be genetically engineered to exhibit specific fat properties or optimized fat content in their meat, tailored to meet diverse applications or dietary preferences. A recent example of such a study is the creation of *LGALS12* knockout piglets using the CRISPR/Cas9 method combined with somatic cell nuclear transfer (SCNT) technology [57]. The *LGALS12* gene has been identified as important for porcine fat deposition. The study revealed that the absence of *LGALS12* suppresses preadipocyte proliferation and affects lipogenesis in porcine intramuscular and subcutaneous adipocytes. Further studies are needed to improve fat traits in pigs through genetic modifications of different loci.

## 4. Materials and Methods

### 4.1. Cell Culture and Induction of Adipogenesis

Mesenchymal stem cells isolated from adipose tissue (AD-MSC) were cultured in Advanced DMEM medium (Gibco, Thermo Fisher Scientific, Waltham, MA, USA) supplemented with 10% FBS (Sigma-Aldrich, Darmstadt, Germany), 5 ng/mL FGF-2 (PromoKine, Heidelberg, Germany), 2 mM L-Glutamine (Gibco), 1 mM 2-mercaptoethanol (Sigma-Aldrich), 1× antibiotic antimycotic solution (Sigma-Aldrich), and 1× MEM NEAA (Gibco) at 37 °C in 5% CO_2_. After the cells reached confluency, adipogenesis was induced using a differentiation medium. The differentiation medium was composed of Advanced DMEM (Gibco), 10% FBS (Sigma-Adrich), 1× antibiotic antimycotic solution (Sigma-Aldrich), 5 ng/mL FGF-2 (PromoKine), 1× Linoleic Acid Albumin (Sigma-Aldrich), 1× ITS Supplement (Sigma-Aldrich), 1 µm Dexamethasone (Sigma-Aldrich), 100 µm Indomethacin (Sigma-Aldrich), and 50 µm IBMX (Sigma-Aldrich). Differentiation was allowed to occur over ten days, with the medium being changed every 24 h.

### 4.2. Monitoring of Lipid Droplet Formation

The accumulation of lipid droplets was monitored every day by visual examination under phase-contrast microscopy (TS100 Eclipse, Nikon, Melville, NY, USA). BODIPY staining was performed by fixing cells with 4% paraformaldehyde in PBS (*w*/*v*) for 10 min at room temperature and washed with PBS three times. The cells were then incubated with BODIPY (Life Technologies, Grand Island, NY, USA) in PBS (2.7 µg/mL) and washed three times in PBS. The nuclei were counterstained with DAPI in Vectashield medium (Vector Laboratories, Newark, CA, USA). The BODIPY fluorescence intensity was measured using a Nikon E600 Eclipse microscope (Melville, NY, USA) and Lucia software version 1.0 (Laboratory Imaging, Prague, Czech Republic) and quantified using ImageJ software version 1.54g (NIH, Bethesda, MD, USA).

### 4.3. gRNA Design and MSC Transfection

gRNAs targeting the 5′-flanking region of the *SREBF1c* gene were designed using the CRISPOR tool (http://crispor.tefor.net/ accessed on 15 January 2022). The sequence of gRNA is shown in Table S1. The gRNA oligos with a BbsI overhang were cloned into the pX330 vector (Addgene #42230, Cambridge, MA, USA) carrying a U6 promoter, an sgRNA scaffold sequence, and the puromycin resistance gene. Then, 2 µg DNA from two plasmids was used for cotransfection into AD-MSCs by nucleofection with a P2 Primary Cell 4D-Nucleofector X Kit (Lonza, Basel, Switzerland) using a 4D Nucleofector Lonza system (Lonza). Transfection efficiency was determined using a control plasmid (pmaxGFP Vector, Lonza). After transfection with plasmids DNA, cells were plated on six-well plates and, after the next 24 h, they were selected using 2 µg/mL puromycin for 48 h. Cells were further cultured to obtain single-cell colonies.

### 4.4. PCR Genotyping of AD-MSC Cells

PCR reactions were performed to screen cell colonies for a deletion in the 5′-regulatory sequence of *SREBF1c*. First, genomic DNA was isolated from single MSC colonies using QuickExtract DNA Extraction Solution (Biosearch Technologies, Petaluma, CA, USA) and from wild-type MSCs using MasterPure Complete DNA & RNA Purification kit (Biosearch Technologies). PCR reactions were performed using *SREBF1c* gRNA1 F: 5′ctgagactgctggggagtgt and *SREBF1c* gRNA3 R: 5′tcaggagcgggctctcac primers. The expected PCR product was 954 bp in unmodified cells and approximately 400 bp in MSC colonies with a deletion in the 5′-regulatory sequence of *SREBF1c*.

### 4.5. Sanger DNA Sequencing

The PCR products were purified using Exonuclease I and FastAP Thermosensitive Alkaline Phosphatase (Thermo Fisher Scientific, Waltham, MA, USA). After the purification, a BigDye Terminator v3.1 Cycle Sequencing kit (Thermo Fisher Scientific) was used to generate fragments that were subsequently filtered using Sephadex G-50 (Sigma-Aldrich). Capillary electrophoresis was run on 3130 Genetic Analyzer (Applied Biosystems, Thermo Fisher Scientific, Waltham, MA, USA), and the resulting chromatograms were analyzed using the SeqMan Pro (DNASTAR) software version 12.2.0 package.

### 4.6. RNA Isolation, cDNA Synthesis, and Real-Time PCR

Total RNA was extracted from AD-MSC cell cultures as well as from subcutaneous and visceral adipose tissues using TriPure Isolation Reagent (Roche Diagnostic, Mannheim, Germany). After quantitative and qualitative analyses with a Nanodrop 2000 (Thermo Scientific) spectrophotometer, 1 µg of RNA was reverse-transcribed into cDNA using a Transcriptor First Strand cDNA Synthesis Kit (Roche). Real-time PCR primers were designed for each transcript to anneal to different exons using the Sscrofa 11.1 reference sequence (Table S2). PCR reactions were performed in triplicate on a Light Cycler 480 II (Roche) instrument with a Light Cycler 480 SYBR Green I Master Kit (Roche). The relative mRNA levels of tested genes were quantified using the second derivative maximum method (Roche), and the results were normalized using the geometric mean of expression of *RPL27* and *PPIA* as reference genes [58,59].

### 4.7. Experimental Control Procedures

All results obtained from CRISPR/Cas9 editing were compared to unedited cells, which served as the control group. To verify the functionality of the CRISPR/Cas9 system, a fluorescent reporter system using a vector containing GFP was employed. In silico prediction tools were utilized to identify potential off-target sites, and only sgRNAs with an extremely low probability of off-target modifications were selected. Two different techniques were used to validate the results: PCR with gel electrophoresis and Sanger sequencing. The experiments were conducted with multiple replicates (*n* = 3). Only cells obtained from single colonies in which the deletion was confirmed by PCR and Sanger sequencing were selected for the next steps of the study—differentiation into adipocytes. Appropriate statistical analyses were performed to evaluate the significance of the results.

### 4.8. Statistical Analysis

The analysis was conducted using IBM SPSS Statistics 28. A significance level of 0.05 was adopted. To compare the results of repeated measurements between the study groups, a MANOVA with repeated measures was used. Sphericity was assessed using Mauchly’s test, and, in the case of non-sphericity, the Greenhouse–Geisser correction was applied to the MANOVA. Due to the small number of measurements, a detailed analysis of the descriptive statistics was also performed, as presented in the tables and graphs. When sphericity assumptions could not be met and MANOVA was not feasible, the t-test was used to compare the data between groups in successive measurements, while the Friedman test was used to compare the results within the same group across multiple measurements. The Shapiro–Wilk test was chosen to assess the normality of the distributions. To compare results between two groups, a non-parametric independent samples test (the Mann–Whitney U-test) was used due to the lack of normal distribution.

## 5. Conclusions

The use of the CRISPR/Cas9 method to generate modified porcine MSCs with a large deletion in the regulatory region of the *SREBF1* gene allowed us to determine the poorly-understood role of the *SREBF1c* isoform as an important factor in the process of adipogenesis in the pig. These findings not only contribute to a deeper understanding of the molecular mechanisms underlying the formation of fat cells in the pig but also open new possibilities for targeted genetic modifications to influence fat deposition in pigs. This could improve meat quality in the pig industry and support the development of porcine models for human obesity research.

## Figures and Tables

**Table 1:** Summary of the results of lipid accumulation (BODIPY) and gene expression analysis in AD-MSC_DEL_ when compare to AD-MSC_WT_. An arrow pointing down (↓) indicates lower expression, an arrow pointing up (↑) indicates higher expression, and an equal sign (=) indicates no difference between the two cell types; - means there was no detection on that day.

	Day 0	Day 2	Day 4	Day 6	Day 8	Day 10
BODIPY	-	↓	↓	↓	↓	↓
*SREBF1a*	=	↓	=	↑	↑	=
*SREBF1c*	↓	↓	↓	↓	↓	↓
*GATA2*	=	=	=	↑	=	=
*CEBPD*	↓	↑	↓	↓	=	=
*CEBPB*	↓	↑	↓	↑	↑	=
*CEBPA*	↑	↓	=	↓	↓	↓
*PPRAG*	↓	↓	↓	↓	↓	↓
*FABP4*	↑	=	=	↓	=	↓

## Data Availability

The data supporting the findings of the study are available from the corresponding author (I.S.) upon reasonable request.

## Supplementary Materials

The following supporting information can be downloaded at https://www.mdpi.com/article/10.3390/ijms252312677/s1.
