# Supplementary material for: Deciphering the Role of the SREBF1 Gene in the Transcriptional Regulation of Porcine Adipogenesis Using CRISPR/Cas9 Editing
Source: Int J Mol Sci. 2024 Nov 26;25(23):12677. doi: 10.3390/ijms252312677 (PMC11641310; doi:10.3390/ijms252312677)
Supplement: Supplementary file 1 [file ijms-25-12677-s001.zip › ijms-3320899-supplementary.pdf]

Supplementary materials

Deciphering the role of the *SREBF1* gene in the transcriptional regulation of porcine adipogenesis using CRISPR/Cas9 editing

Mehmet Onur Aksoy,[1, †] Adrianna Bilinska,[1, †] Monika Stachowiak,[1] Tatiana Flisikowska,[2] Izabela Szczerbal[1,*]

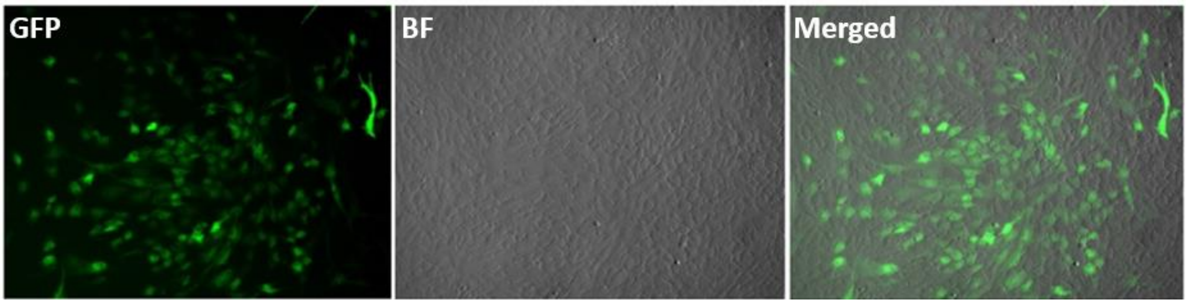

Figure S1. GFP expression in AD-MSC after nucleofection with control plasmid.

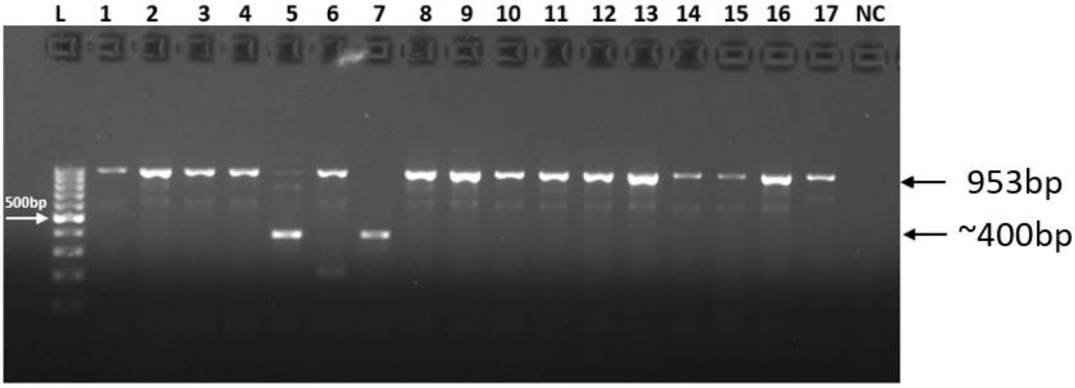

Figure S2. PCR detection of a deletion in the 5'-regulatory sequence of *SREBF1c* in single AD-MSC colonies. The expected PCR product size was 953 bp in unmodified MSC (WT), but shorter (~400 bp) in the targeted MSC colonies (lines 5 and 7). L: 100–1000 bp DNA ladder; NC: negative control (sample with no DNA template).

Figure S3. Relative mRNA levels of *SREBF1a* and *SREBF1c* isoforms in the subcutaneous and visceral adipose tissue of wild type pigs.

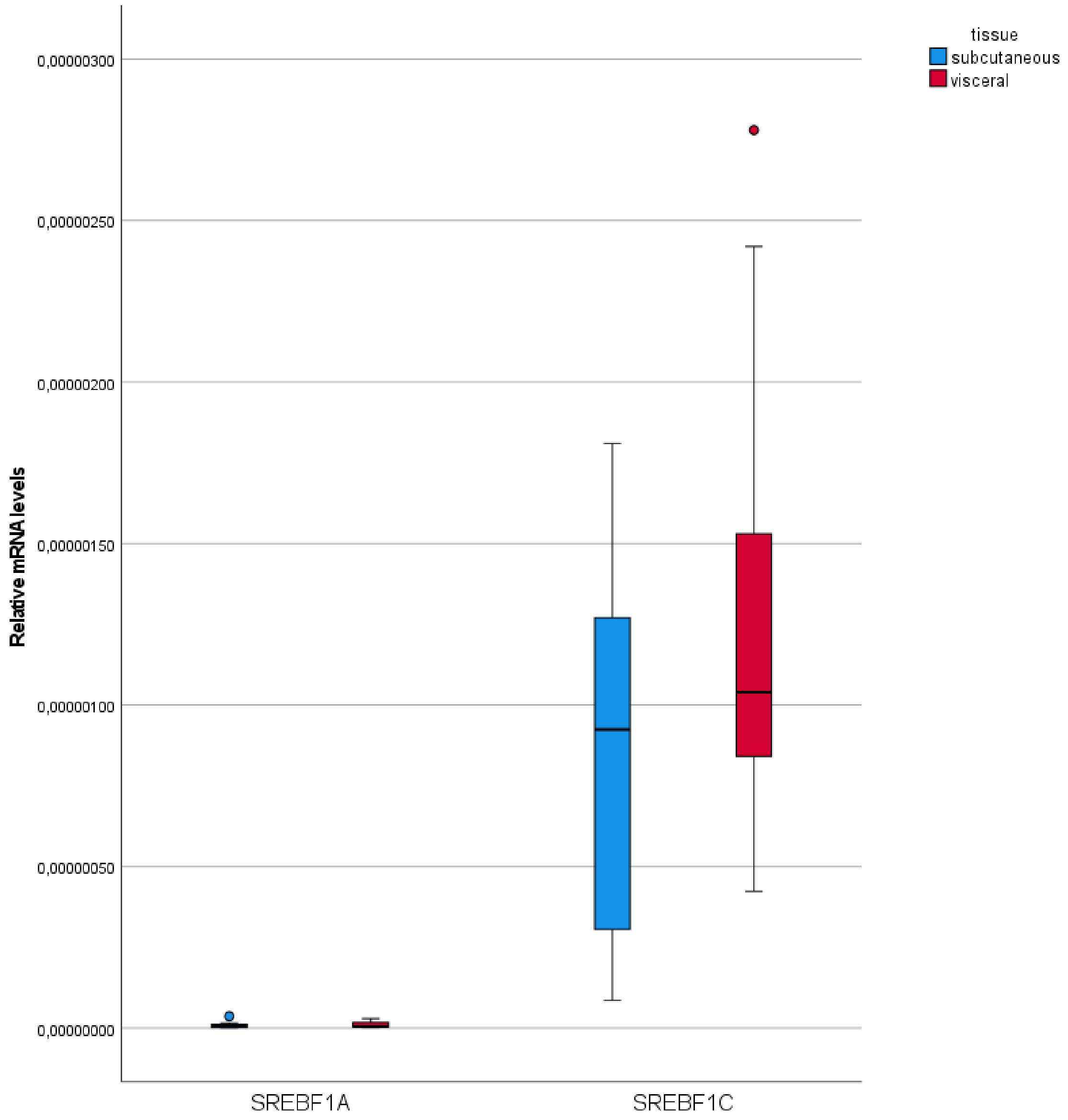

Table S1. gRNAs/primers directed against the 5'flanking region of *SREBF1c* gene.

gRNA	Name	Sequence
gRNA1	SREBF1c_g1_F	CACCGtacttgtccccgtcgatgca
	SREBF1c_g1_R	AAACtgcatcgacggggacaagtaC
gRNA3	SREBF1c_g3_F	CACCGaccatggactgcacgttcga
	SREBF1c_g3_R	AAACtcgaacgtgcagtccatggtC

Table S2. List of primer pairs used in RT-qPCR.

Gene	Primer sequence	Product size (bp)	Annealing temperature (°C)	Genbank accession number
*RPL27**	F: 5` GCAAAGCGGTCATCGTAAA<br>R: 5` CTTGTGGGCATGAGGTGAT	190	60	NM_001097479.1
*PPIA**	F: 5 `CACAAACGGTTCCCAGTTTT<br>R: 5 `TGTCCACAGTCAGCAATGGT	171	60	XM_021078519.1
PPARG	F: 5` GCATCAGCTCTGTGGACCTG<br>R: 5` GATCAGCTCTCGGGAATGGG	132	60	XM_005669783
CEBPA	F: 5` CGTGAGCGCAACAACATCG<br>R: 5` CTCAGTTGTTCCACCCGCTT	131	60	NC_010448
FABP4	F: 5` TTCAAATTGGGCCAGGAAT<br>R: 5` ATTCTGGTAGCCGTGACACC	191	60	NM_001002817
CEBPD	F: 5` TGGTTGCTGTTGAAGAGGTCA<br>R: 5` CCATCGACTTCAGCGCCTAC	97	60	XM_005663091.2
CEBPB	F: 5` TACTACGAGGCGGACTGCTT<br>R: 5` TCCAGGTATGGGCTGAAGTC	152	60	NM_001199889.1
GATA2	F: 5` CTCCAGCTTCACCCCTAAG<br>R: 5` CCCGTTCATCTTGTGGTACAG	157	60	XM_021068525.1
SREBF1c	F: 5` TCGAAGACATGCTTCAGCTC<br>R: 5` GGAGCTCATGGTGGAAGGAG	155	60	XM_021066226.1
SREBF1a	F: 5` ACAGAGGCAGCCTTGGAG<br>R: 5` GTCGCTGTCTTGGTTGTTGA	108	60	NM_214157.1

* *reference gene*
